# Development of Nanofiber Mats Impregnated With *Ferula assa-foetida* Essential Oil Nanogel for Antibacterial Wound Care

**DOI:** 10.1155/ijbm/1436095

**Published:** 2025-06-10

**Authors:** Mahmoud Osanloo, Sahar Fereydonpour, Abbas Abdollahi, Mojdeh Safari

**Affiliations:** ^1^Department of Medical Nanotechnology, School of Advanced Technologies in Medicine, Fasa University of Medical Sciences, Fasa, Iran; ^2^Student Research Committee, Fasa University of Medical Sciences, Fasa, Iran; ^3^Department of Microbiology, School of Medicine, Fasa University of Medical Sciences, Fasa, Iran; ^4^Finetech in Medicine Research Center, School of Medicine, Iran University of Medical Sciences, Tehran, Iran

**Keywords:** alternative therapeutics, herbal antibacterial agents, nanofiber mats

## Abstract

Bacterial infections pose significant threats to human health, and the rising issue of antibiotic resistance necessitates exploring alternative therapeutic strategies. In this study, *Ferula assa-foetida* essential oil (EO) as an herbal medicine was first analyzed using gas chromatography-mass spectrometry (GC-MS). Polycaprolactone-gelatin nanofibers were then prepared via electrospinning. Biological efficacies (antioxidant and antibacterial properties) of nanofibers impregnated with the nanogel containing the EO were finally investigated. As a result, the five major identified compounds were ethyl trifluoromethyl disulfide (33.6%), β-pinene (15.1%), δ-3-carene (6.6%), dihydro-β-agarofuran (6.0%), and γ-eudesmol (5.5%). Nanogel was developed from a primary nanoemulsion, with a 55 ± 7 nm droplet size and a zeta potential of −31 ± 2 mV. Nanofibers with a hydrophobic surface (contact angle (*θ*) 107°) impregnated with the nanogel demonstrated remarkable antibacterial efficacy, inhibiting the growth of *Escherichia coli* and *Staphylococcus aureus* by nearly 100% and *Pseudomonas aeruginosa* by 90%. These findings suggest that the developed formulation has the potential to serve as an effective antibacterial wound dressing, warranting further investigation.

## 1. Introduction

Bacteria play a dual role in human health, acting both as friends and foes. Among the most notorious pathogens, *Escherichia coli*, *Pseudomonas aeruginosa*, and *Staphylococcus aureus* pose significant challenges to human well-being [1, 2]. The escalating global issue of antibiotic resistance has intensified the search for alternative therapeutic approaches, leading researchers to explore the potential of plant-based antimicrobial agents [3, 4].

Plants have long served as a rich source of bioactive compounds with antimicrobial properties, offering promising avenues for developing novel treatments. Herbal antibacterial agents, particularly essential oils (EOs), present diverse chemical structures and often exhibit synergistic effects, making them less prone to resistance development than conventional antibiotics [5, 6]. Moreover, they are generally considered safer with fewer side effects, addressing a critical concern in modern medicine [7, 8]. *Ferula assa-foetida*, also known as asafetida, has a long history of medicinal use [9]. Studies have shown that *F. assa-foetida* has potential anticancer properties, particularly in treating colorectal cancer [10]. It also has anthelmintic properties, making it effective against parasitic diseases such as dicrocoeliasis and fascioliasis [11]. Furthermore, its antibacterial effects against various bacteria have been reported [12, 13]. For instance, a study analyzing the EO from *F. assa-foetida* collected from the Neishabour mountains found that it exhibited antibacterial activity against both Gram-positive (*Staphylococcus epidermidis*, *Bacillus subtilis*, and *S. aureus*) and Gram-negative (Klebsiella pneumonia, *E. coli*, and *P. aeruginosa*) bacteria, with minimum inhibitory concentrations ranging from 12 to 24 mg/mL [14].

Modern drug delivery strategies are essential to improve the effectiveness of such herbal compounds. In the pursuit of herbal antibacterial solutions, nanogels have emerged as a groundbreaking delivery platform, offering distinct advantages in enhancing the efficacy of these herbal agents [15, 16]. Nanogels are nanoscale hydrogel particles that can precisely encapsulate and deliver bioactive compounds. One of the critical advantages of nanogels is their ability to protect herbal antibacterial agents from degradation and optimize their release kinetics. This targeted delivery ensures a sustained and controlled release of the active compounds, maximizing therapeutic effects while minimizing potential side effects [17, 18]. Additionally, nanogels can improve the bioavailability of herbal compounds, enhancing their absorption and distribution in the body. This allows for lower dosage requirements, reducing the risk of toxicity and improving patient compliance. The nanogel structure enables easy modification, allowing researchers to tailor their properties for specific applications, such as sustained release or site-specific targeting [19, 20].

Beyond delivery, the physical scaffold for application also plays a crucial role, especially in wound healing. Nanofiber (NF) mats have emerged as a promising material for wound dressing applications due to their unique properties and advantages. The electrospinning process used to create these mats allows for producing fibers with diameters in the nanometer range, resulting in mats with a high surface area-to-volume ratio [21]. This high surface area facilitates efficient gas exchange, vital for wound healing, ensuring oxygen can reach the wound site while removing carbon dioxide and other waste. At the same time, the tiny pores of NF mats prevent the entry of pathogens and secondary infections [22, 23]. Polycaprolactone (PCL), a synthetic and biodegradable polymer, is a well-known biomaterial frequently utilized in various biomedical applications due to its good mechanical strength, biocompatibility, and surface bioactivity [24]. However, the hydrophobic nature of PCL and its slow biodegradation rate limit its applications. Gelatin, a natural hydrophilic biopolymer derived from the hydrolysis of collagen, is known for creating a moist wound environment, essential for wound healing [25]. PCL–gelatin NFs have been shown to improve thermal stability and mechanical properties compared to pure gelatin and PCL, and they have demonstrated promising wound-healing properties [26, 27]. Moreover, PCL–gelatin NFs are particularly suited for wound healing due to their biocompatibility, biodegradability, and ability to maintain a moist environment—key factors in promoting granulation tissue formation and epithelialization [28, 29].

While direct incorporation of EOs into electrospun NFs may seem straightforward, challenges such as volatility and incompatibility with hydrophilic polymers often lead to inefficient loading and rapid release. In contrast, impregnating a nanogel-encapsulated EO onto NF mats ensures stability, controlled release, and synergistic mechanical benefits. Furthermore, unlike conventional gauze, NF mats provide a high surface area, barrier function against pathogens, and enhanced bioadhesion, making them superior for sustained drug delivery in wound care [30, 31].

In this study, a nanogel encapsulating *F. assa-foetida* EO was developed for the first time. This nanogel was then physically impregnated into electrospun PCL–gelatin NF mats, widely recognized as a model for wound dressings. The antibacterial activity of the resulting composite material was evaluated against *E. coli*, *P. aeruginosa*, and *S. aureus*, aiming to assess its potential in modern wound care management.

## 2. Materials and Methods

### 2.1. Materials

Materials used in this study included commercially acquired carboxymethyl cellulose (CMC), PCL granules, gelatin powder sourced from Sigma-Aldrich (USA), and Tween 80 obtained from Merck Chemicals (Germany). The *F. assa-foetida* EO was procured from Tabib Daru Co. (Iran), while bacterial strains, including *E. coli* (ATCC 25922), *P. aeruginosa* (ATCC 27853), and *S. aureus* (ATCC 25923), were provided by the Pasteur Institute of Iran.

### 2.2. Identification of Compounds in *F. assa-foetida* EO via GC-MS Analysis

The *F. assa-foetida* EO analysis was carried out using an Agilent GC-MS system, consisting of a 6890 gas chromatography coupled with a 5975 network mass selective detector, as detailed in a prior study. Separations occurred on an HP-5MS silica fused column with specific parameters: a column temperature program initiated at 40°C, increased at a rate of 3°C·min^−1^–250°C, and held at this temperature for 60 min. The injection port and detector temperatures were set at 250°C and 230°C, respectively. Operational conditions included helium as the carrier gas, split flow at 25 mL/min, septum purge at 6 mL/min, and a 1 mL/min column flow rate. Mass spectra were acquired in full scan mode with 70 eV ionization energy and a scanned mass range of 50–550 m/z. Identifying *F. assa-foetida* EO constituents involved determining retention indices by comparing them with a homologous series of C6–C27 normal alkanes. Retention indices were calculated based on a standard mixture of n-alkanes, and identification was confirmed by comparing mass spectra with the Wiley7n.l MS computer library. Linear temperature-programmed retentions were determined from the gas chromatogram using interpolation. The quantitative determination of compounds in the EO was accomplished through peak area normalization in the *F. assa-foetida* EO analysis.

### 2.3. Preparation of Characterizations of PCL–Gelatin NFs

Gelatin powder and PCL granules (3%:12% w/v) were first dissolved in hexafluoroisopropanol overnight, at 2000 rpm, room temperature. The solution was filled in a 10 mL syringe connected to a blunted needle (18 g) and in a syringe pump of the electrospinning machine (Fanavaran Nano-Meghyas, Iran). A thin layer of aluminum foil was wrapped around the cylindrical collector to separate the formed NFs; the collector was rotated (100 rpm) during the preparation of NFs. Besides, different values for each instrumental factor, including injection rate (0.4, 0.6, and 0.8 mL/h), applied DC voltage between needle and collector (10, 15, and 20 kV). Distance between needle and collector (80, 100, and 120 mm) was screened to obtain beadles NFs with nanosize diameter. Note that optimum NFs were obtained by injection rate of 0.8 mL/h, voltage of 20 kV, and distance of 120 mm.

NFs were punched and coated with gold vapor (sputtering coater, Q150R-ES, Quorum Technologies, UK) before subjecting to an SEM instrument for investigating morphology and size (Scanning Electron Microscopy, Vega 3, TESCAN, Czech Republic). Moreover, FTIR spectroscopy (Bruker, Tensor II, Germany) was performed to detect different functional groups present in the pure components and prepared NFs. Before FTIR analysis, the samples (PCL powder, gelatin powder, and PCL–gelatin NFs) were ground with KBr and compressed into a thin wafer using hydraulic pressure. Finally, the FTIR spectra were collected under transition mode in the 400–3000 cm^−1^ range. Meanwhile, the contact angle measuring device (CA-500A, Sharif Solar Co. Iran) was used to measure the hydrophobicity of the NFs' surface. Five microliters of deionized water were poured on the surface, and its image was taken. The contact angle of the water droplet and the surface was measured with the help of the software. Contact angles higher than 90° are considered hydrophobic.

### 2.4. Preparation of Characterizations of Nanogel Containing *F. assa-foetida* EO

*F. assa-foetida* EO (2% w/v) and Tween 80 (2.5% w/v) were mixed at 2000 rpm for 5 min at room temperature. Distilled water was then dropwisely added, and the mixture was stirred for another 60 min to obtain a primary nanoemulsion containing *F. assa-foetida* EO. Another nanoemulsion was prepared similarly without *F. assa-foetida* EO. Droplet size, droplet size distribution (SPAN), and Zeta potential values of samples were investigated using DLS type apparatus.

Moreover, both nanoemulsions containing/without *F. assa-foetida* EO were gelified using CMC. CMC powder was weighed (3.5% w/v), added to the nanoemulsions, and stirred for 18 h at room temperature to hydrate and complete the gelation process. The viscosity of nanogel containing *F. assa-foetida* EO and nanogel without the EO (NGel(-oil)) at different shear rates (0.1–100 1/s) were scanned using a rheometer machine (MCR-302, Anton Paar rheometer Company, Austria). ATR-FTIR was used to confirm the successful loading of the EO in the nanogel. Spectra of *F. assa-foetida* EO, nanoemulsion without EO, nanoemulsion containing *F. assa-foetida* EO, nanogel without EO (NGel(-oil)), and nanogel containing *F. assa-foetida* EO were recorded at 400–3000 cm^−1^.

### 2.5. Impregnating Nanogel on NFs and Investigation of Biological Assays

**Figure 1:**
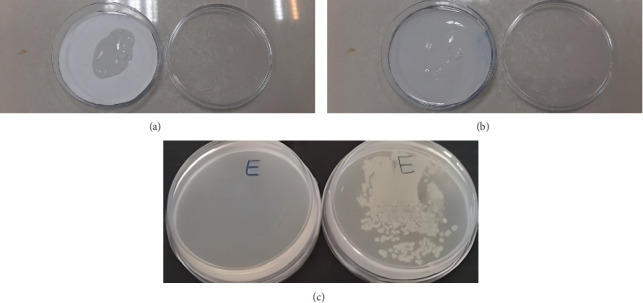
(a) Impregnating nanogel on PCL–gelatin NFs, (b) adding bacterial suspension on impregnated NFs with nanogel, (c) comparing bacterial growth in a treated plate with impregnated NFs with nanogel and control group.

The PCL–gelatin NFs were cut into circles with a diameter of 5 cm; both sides were sterilized using UV irradiation for 60 min in Class II Biosafety Cabinet. Subsequently, varying amounts of nanogel (1000, 500, and 250 mg), as well as 1000 mg of NGel(-oil), were applied to the NFs (Figure 1(a)). The impregnation process was carried out under sterile conditions near two open flames. These samples were used in antioxidant and antibacterial assays, as follows.

A DPPH assay was used to investigate the antioxidant effects. First, 5 cm plates containing as-prepared impregnated NFs with nanogel and NGel(-oil) were ready. A control group and two negative control groups were also considered. The control group was not treated, and negative control groups were treated with 1000 mg of NGel(-oil) and a piece of NFs with a diameter of 5 cm. After that, 4 mL of DPPH solution 0.2 mM (dissolved in absolute ethanol) was added. The plates were incubated in a dark place for 30 min, and the absorbance of their supernatants was read at 517 nm using a plate reader. Antioxidant effects were calculated by OD control − OD sample/OD control × 100.

The AATCC100 was used to investigate the antibacterial effects. First, 5 cm plates containing as-prepared impregnated NFs with nanogel and NGel(-oil) were ready. A control group and two negative control groups were also considered. The control group was not treated, and negative control groups were treated with 1000 mg of NGel(-oil) and a piece of NFs with a diameter of 5 cm. After that, 4 mL of each bacterial suspension (2 × 10^5^ CFU/mL), including *E. coli, P. aeruginosa*, and *S. aureus,* was added (Figure 1(b)). The plates were incubated for 24 h in a shaking incubator (90 rpm) at 37°C, and after that, 10 μL of supernatant of each plate was cultured on the Mueller Hinton agar plates and incubated for 24 h. Bacterial growth inhabitation was calculated using CFU control − CFU sample/CFU control  ×  100 (Figure 1(c)).

An explanation regarding the calculation of concentrations is noted. The nanogel contained 2% or 20,000 μg/mL of *F. assa-foetida* EO. In the bacterial test, 4 mL of bacterial suspension was exposed to impregnated NFs with 1000, 500, and 250 mg of nanogel. Therefore, it can be said that bacteria were exposed to impregnated NFs with nanogels containing 5000, 2500, and 1250 μg/mL of *F. assa-foetida* EO, respectively. Besides, bacteria in the negative control group were exposed to 250 mg/mL of impregnated NFs with NGel(-oil).

All experiments were done in triplicates, and the results are mean ± standard deviations. One-way ANOVA and Tukey post hoc test were used to compare control group results with samples. GraphPad prism was used to draw charts, and *p* < 0.05 was considered a significant difference.

## 3. Results

From Table 1, ethyl trifluoromethyl disulfide (33.6%), β-pinene (15.1%), δ-3-carene (6.6%), dihydro-β-agarofuran (6.0%), and γ-eudesmol (5.5%) were identified as five major compounds in *F. assa-foetida* EO using GC-MS analysis.

**Figure 2:**
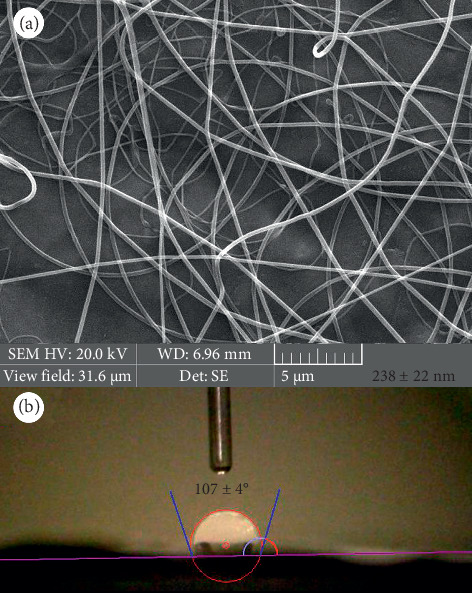
(a) SEM image of PCL‐gelatin NFs and (b) water contact angle with the NFs.

Smooth and beadles PCL–gelatin NFs with a mean diameter of 238 ± 22 nm were prepared (Figure 2(a)). An angle of 107 ± 4° is observed between the water drop and the NFs surface in Figure 2(b), which indicates that the surface is hydrophobic.

**Figure 3:**
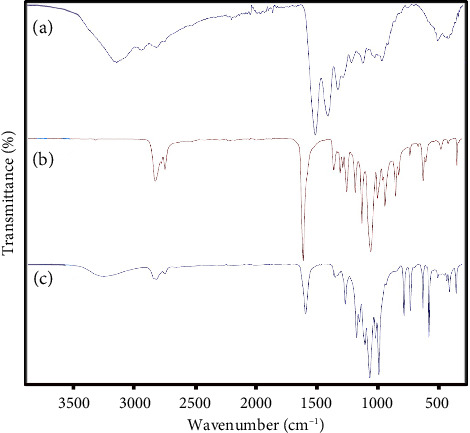
FTIR spectra of (a): PCL granules, (b): gelatin powder, and (c): PCL–gelatin NFs.

FTIR spectra of PCL granules, gelatin powder, and PCL–gelatin NFs are shown in Figure 3. In the spectra of PCL (Figure 3(a)), the sharp peak at 1722 cm^−1^ is attributed to the stretching vibration of the carbonyl (-C=O) group. The bands at 2943 and 2856 cm^−1^ are related to the symmetric and asymmetric stretching vibration of -CH_2_-. The FTIR peak at 1292 cm^−1^ is associated with C-O and C-C stretching vibration in PCL. In addition, peaks located at 1236 and 1164 cm^−1^ are common due to C-O-C symmetric and asymmetric stretching [32, 33]. The spectrum of pure gelatin (Figure 3(b)) exhibits the bands at 1628, 1523, and 1238 cm^−1^ assigned to the stretching of amides I, II, and III, respectively. The peaks at around 3271 and 2945 cm^−1^ confirm the presence of O-H and CH2 groups in the gelatin structure, respectively [34, 35]. The prominent characteristic peaks in the spectrum of pure PCL and gelatin were observed in the electrospun PCL–gelatin NFs (Figure 3(c)), indicating that the obtained NFs contain both PCL and gelatin. Some changes in the position and intensity of peaks were recognized. For example, the absorption peak at 1722 cm^−1^ related to the carbonyl (C=O) group in pure PCL was shifted to 1704 cm^−1^ with lower intensity in the spectrum of PCL–gelatin NFs, indicating the interaction between gelatin and PCL. Notably, a weak absorption peak at 1238 cm^−1^ for amid III in gelatin is hidden by the asymmetric vibration of C-O-C in PCL. Similar observations were also reported in the literature [35–37].

**Figure 4:**
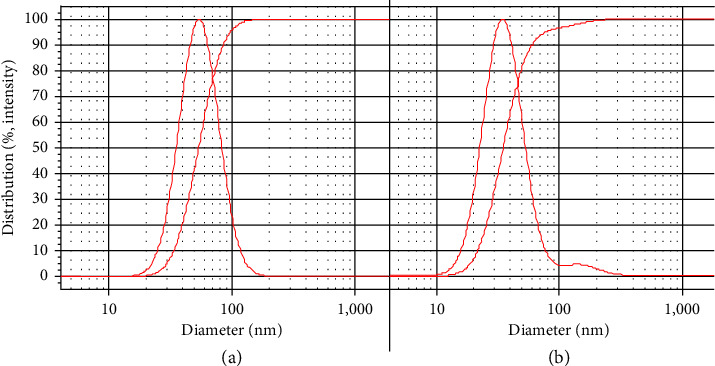
DLS profiles of primary nanoemulsions (a): containing *F. assa-foetida* EO and (b): without EO.

**Figure 5:**
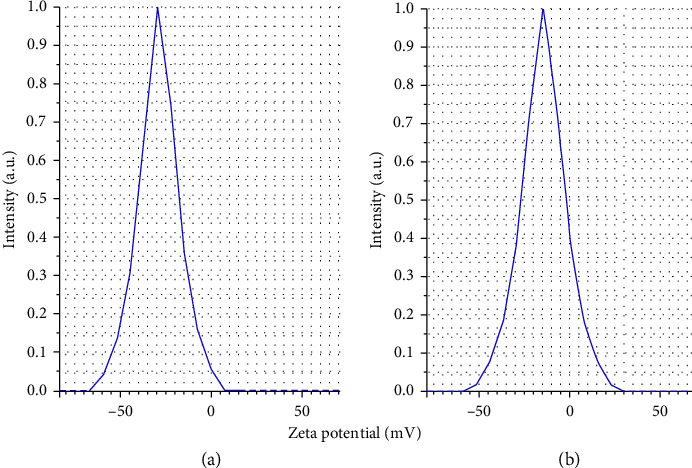
Zeta potential profiles of primary nanoemulsions (a): containing *F. assa-foetida* EO and (b): without EO.

DLS profiles of primary nanoemulsion containing *F. assa-foetida* EO and nanoemulsion without EO (NGel(-oil)) are shown in Figures 4(a) and 4(b). Droplet sizes were obtained as 55 ± 7 and 35 ± 13 nm, and SPAN values were obtained as 0.94 and 1.1. Besides, as depicted in Figures 5(a) and 5(b), their zeta potential was −31 ± 2 and −19 ± 2 mV.

**Figure 6:**
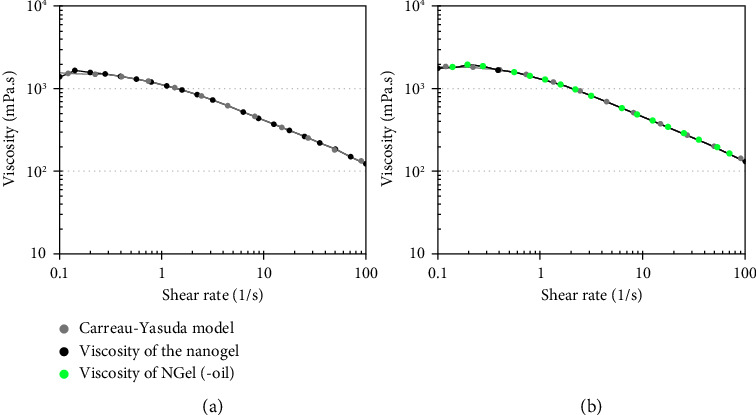
Viscosity curves of nanogels (a): containing *F. assa-foetida* EO and (b): without EO (NGel(-oil)).

Viscosity curves of nanogels containing *F. assa-foetida* EO and nanogel without EO (NGel(-oil)) are illustrated in Figures 6(a) and 6(b). Their viscosity matches the Carreau-Yasuda model, a well-known regression for non-Newtonian fluids.

**Figure 7:**
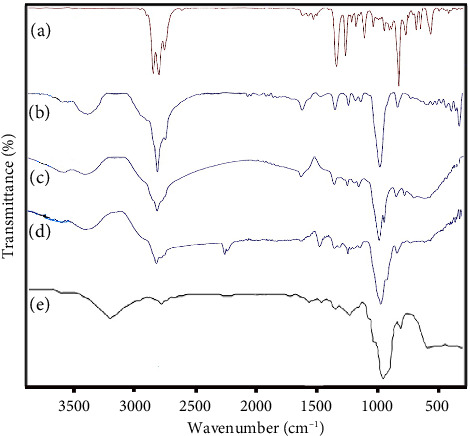
ATR-FTIR spectra of (a): *F. assa-foetida* EO, (b): nanoemulsion without EO, (c): nanoemulsion containing *F. assa-foetida* EO, (d): nanogel without EO (NGel(-oil)), and (e): nanogel containing *F. assa-foetida* EO.

Moreover, the ATR-FTIR spectra of the nanogels and their respective components are displayed in Figure 7. The spectrum of *F. assa-foetida* EO (Figure 7(a)) revealed several functional group signals, including distinct bands at 3069 and 2963 cm^−1^, which correspond to the stretching vibrations of phenolic O−H and aromatic C−H, respectively. Additional peaks observed at 2916, 2872, 1376, and 1219 cm^−1^ were associated with alkane C–H stretching modes. The strong absorption near 1730 cm^−1^ was attributed to carbonyl (C=O) stretching, while the band at 875 cm^−1^ indicated the presence of C–H out-of-plane bending in alkenes. The C–C stretching vibration of the aromatic ring appeared around 1447 cm^−1^, and the C–O–C stretching was found near 1261 cm^−1^. In the spectrum of the primary nanoemulsion without EO (Figure 7(b)), two bands at 2924 and 2858 cm^−1^ correspond to the asymmetric and symmetric stretching of methylene groups (-CH_2_-), respectively. A prominent peak at 1733 cm^−1^ was attributed to ester carbonyl stretching, and the broad absorption around 3498 cm^−1^ indicated hydroxyl group vibrations [38].

For the *F. assa-foetida* EO-loaded nanoemulsion (Figure 7(c)), peaks from both the EO and the blank nanoemulsion were observed, although some shifts in intensity and wavenumber occurred, suggesting successful incorporation of the EO into the nanoemulsion system. These observations are consistent with previous findings [39–41].

In the nanogel without EO (NGel(-oil)), Figure 7(d)), the broad peak spanning 3508–3698 cm^−1^ was attributed to the O–H stretching vibrations. Other noticeable bands included 2922, 1581, and 1461 cm^−1^, corresponding to C–H stretching, C=O stretching, and CH_2_ groups, respectively. The broad band from 1080 to 1252 cm^−1^ was assigned to ether (-O-) stretching vibrations. Finally, the spectrum of the EO-loaded nanogel (Figure 7(e)) exhibited prominent bands related to the EO, as well as the signature peaks of CMC and Tween 80, confirming the successful encapsulation of the EO within the nanogel matrix [42].

**Figure 8:**
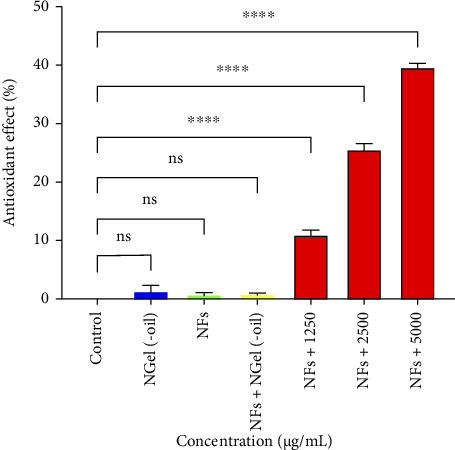
Antioxidant effects of nanogel without EO (NGel(-oil)), PCL–gelatin NFs, impregnated NFs with 250 mg/mL of NGel(-oil) (NFs + NGel(oil)), and impregnated NFs with different amounts of nanogel containing the EO. ns: *p* > 0.05 and ^∗∗∗∗^: *p* < 0.0001.

No significant differences (*p* > 0.05) were observed between the antioxidant effects of NGel(-oil) and NFs and the control group, as shown in Figure 8. However, by impregnating 1250, 2500, and 2500 μg/mL of the nanogel on the NFs, some percent of antioxidant effects are observed, 11 ± 0.8, 26 ± 1.1, and 40 ± 0.8.

**Figure 9:**
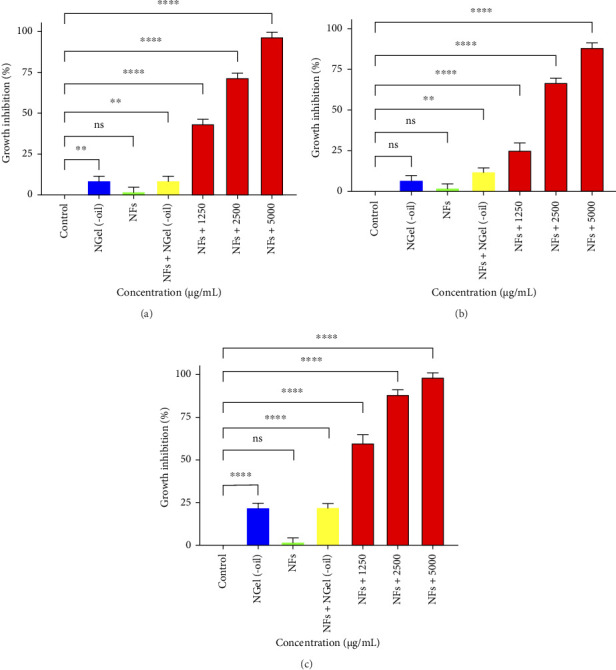
Antibacterial effects of nanogel without EO (NGel(-oil)), PCL–gelatin NFs, impregnated NFs with 250 mg/mL of NGel(-oil) (NFs + NGel(oil)), and impregnated NFs with different amounts of nanogel containing the EO against (a): *E. coli*, (b): *P. aeruginosa*, and (c): *S. aureus* ns: *p* > 0.05, ^∗∗∗∗^: *p* < 0.01, and ^∗∗∗∗^: *p* < 0.0001.

Antibacterial effects of NGel(-oil), NFs, NFs + NGel(oil), and impregnated NFs with 1250, 2500, and 5000 μg/mL of the nanogel against *E. coli*, *P. aeruginosa*, and *S. aureus* are shown in Figures 9(a), 9(b), and 9(c). The best efficacy is related to impregnated NFs with nanogel containing 5000 μg/mL of the *F. assa-foetida* EO; 97 ± 3%, 88 ± 2%, and 98 ± 2.5% of the growth of the bacteria were inhibited. Besides, impregnated NFs with 250 mg/mL of NGel(-oil) showed some degree of bacteria growth inhibitory effects; 8 ± 2%, 7 ± 3%, and 22 ± 3%.

## 4. Discussions

Bacteria are still a health problem, even in developed countries. *E. coli*, commonly found in the intestines, typically maintains a symbiotic relationship with humans. However, certain strains of *E. coli* can lead to severe infections, causing gastrointestinal distress, urinary tract infections, and, in extreme cases, life-threatening complications [43, 44]. *P. aeruginosa*, an opportunistic pathogen, is particularly troublesome in healthcare settings, where its ability to exploit immunocompromised patients can result in pneumonia, urinary tract infections, and wound infections. Its intrinsic antibiotic resistance further complicates treatment efforts [45, 46]. Meanwhile, *S. aureus*, often residing on the skin and in nasal passages, can cause skin infections, pneumonia, and more menacingly, methicillin-resistant *S. aureus* (MRSA) strains pose a grave threat due to their resistance to multiple antibiotics [47, 48].

This study used *F. assa-foetida* EO as an antibacterial agent. While the precise molecular mechanisms underlying its antibacterial activity remain unclear in the current literature, it can be inferred that it disrupts bacterial cell membranes, interferes with essential cellular processes, or inhibits key enzymes necessary for bacterial survival [49, 50]. However, the mechanism of antibacterial effects of some other EOs has been reported. *Lemon verbena* EO was found to destroy the integrity of cells, prevent biomembrane formation, and cause cell death by enhancing the permeability of the cell membrane and leakage of contents [51]. EOs, such as *Origanum vulgare* and *Foeniculum vulgare*, demonstrated inhibitory effects on bacterial growth and reduced biofilm formation and activity, possibly through epigenetic changes [52]. Clove EO and its main component, eugenol, disrupted the *S. aureus* cell wall and membrane, leading to cell apoptosis due to oxidative stress. Eugenol also inhibited bacterial DNA cleavage and combined with specific enzymes involved in bacterial metabolism [53]. EOs have been shown to inhibit biofilm formation and efflux pumps [54].

This study employed CMC as a thickening agent to gelify the primary nanoemulsion containing *F. assa-foetida* EO. CMC has been studied in various nanocomposite formulations for its antimicrobial properties. CMC-based nanocomposites have demonstrated potent activity against bacterial strains such as *E. coli* and *S. aureus*, particularly when interacting with bacterial membranes [55]. Studies have shown that incorporating different nanoparticles into CMC, such as TiO_2_ and silver nanoparticles, enhances its antimicrobial efficacy [56]. In one study, hydrogel nanocomposite films based on CMC crosslinked with N,N′‐methylene‐bis‐acrylamide and loaded with TiO_2_ nanoparticles exhibited enhanced antimicrobial activity [57]. This activity is often attributed to membrane disruption, compromising bacterial integrity and function [58, 59]. Besides, CMC has also enhanced antibacterial activity via synergistic effects; this synergy may involve a combination of effects on membrane permeability, efflux pump activity, and other bacterial processes [60, 61].

This study optimized a PCL–gelatin NFs mat, a well-established wound dressing platform, using an electrospinning device (0.8 mL/h, 20 kV, 120 mm). These parameters enabled the fabrication of NFs with uniform morphology, which aligns with previous research demonstrating the importance of voltage and tip-to-collector distance in achieving optimal fiber formation, particularly for mixed hydrophobic/hydrophilic polymer systems. The flow rate of 0.8 mL/h prevented bead formation, a common issue caused by imbalanced solution supply and solvent evaporation. Such control over electrospinning conditions enhanced the structural uniformity and improved the mechanical and biological functionality of the dressing [26, 62].

The porous structure of these mats provides an ideal moist environment that promotes cell migration and proliferation, key factors in the wound healing process [28]. Gelatin is used as a component in wound dressings to enhance wound healing properties; it improves the wettability of wound dressings, allowing for better absorption of wound exudates [63]. Adequate humidity promotes a moist wound environment, which is essential for cell migration, angiogenesis, and collagen synthesis, as well as prevents wound desiccation and promotes the formation of granulation tissue [64]. PCL is used in wound healing applications due to its biocompatibility and biodegradability. It provides mechanical strength and stability to wound dressings, providing proper wound protection and support [65, 66].

Interestingly, the proposed system in the current study, i.e., impregnated PCL–gelatin NFs with nanogel containing 5000 μg/mL of *F. assa-foetida* EO, was able to stop the growth of bacteria *E. coli* and *S. aureus* almost 100%, and 90% of *P. aeruginosa* growth. This level of efficacy is noteworthy, especially when compared to other EO-based nanogels. For instance, lavender EO nanogel (5000 μg/mL) exhibited ∼50% growth inhibition against S. aureus, while geranium EO nanogel (5000 μg/mL) showed ∼20% inhibition [67]. Nanogel containing *Cuminum cyminum* EO (5000 μg/mL) showed more than 90% and 80% efficacy against *P. aeruginosa* and *S. aureus* [68].

The PCL–gelatin NF matrix not only serves as a physical protective barrier but also enhances the practicality of the nanogel by providing structural support and preventing aggregation. This synergistic interaction ensures uniform distribution of the EO, prolonged contact with the wound bed, and protection against rapid clearance by exudates. Moreover, the antibacterial and antioxidant properties of the nanogel-impregnated NFs address two major barriers to wound healing: infection and oxidative damage. By neutralizing reactive oxygen species and preventing bacterial colonization, the system promotes an optimal healing environment, a conclusion supported by prior research linking these properties to accelerated tissue regeneration [53, 69].

While this study establishes the antibacterial and antioxidant foundation of the proposed formulation, further studies are warranted to evaluate its full therapeutic potential. Future research will include in vitro cell-based assays (e.g., fibroblast proliferation and scratch assays) and in vivo assessments using rodent wound models. In addition, cytotoxicity and biocompatibility studies will be essential to confirm its safety and feasibility for clinical translation.

## 5. Conclusion

This study successfully incorporated *F. assa-foetida* EO on the PCL–gelatin NFs mats via a nanogel delivery system. The hybrid NF-nanogel system developed here leverages the structural advantages of PCL–gelatin mats to overcome the limitations of traditional EO delivery methods, offering a promising strategy for antibacterial wound care. The impregnated NFs exhibited significant antibacterial efficacy against *E. coli*, *P. aeruginosa*, and *S. aureus*. The NFs also exhibited dose-dependent antioxidant effects. These findings highlight the potential of this novel formulation as an effective and sustainable antibacterial wound dressing. Further, in vivo studies are warranted to validate its clinical efficacy.

## Figures and Tables

**Table 1:** Compounds with a higher portion than 1% that identified in the *F. assa-foetida* EO.

Ret time	Compound	Area	%	RI
8.9	β-Pinene	11,432,098,128	15.1	603
9.2	β-Myrcene	1,368,982,577	1.3	614
9.6	α-Phellandrene	1,517,089,666	1.5	630
10.0	2,3,5-Trimethylthiophene	1,600,161,378	1.6	643
10.6	δ-3-Carene	4,719,429,793	6.6	670
11.1	*cis*-Ocimene	3,602,558,116	3.5	687
11.6	β-Ocimene	5,321,903,918	5.2	706
14.2	2,3,4,5-Tetramethylthiophene	1,058,425,339	1.0	771
18.2	Ethyl trifluoromethyl disulfide	28,287,639,983	33.6	858
18.9	Disulfide, bis(1-methylpropyl)	1,414,501,461	1.4	871
20.6	5,6-Dihydro-2-methyl-1,4-dithiine-3-carboxylic acid	2,966,449,925	2.9	904
26.2	7-Tetradecene	1,197,285,424	1.2	1010
27.8	Thiazolidine	2,827,241,765	2.8	1041
28.4	α-Selinene	2,245,194,784	2.2	1052
28.8	α-Humulene	1,300,122,886	1.3	1059
30.6	Dihydro-β-agarofuran	4,147,513,569	6.0	1094
31.2	α-Amorphene	1,107,232,701	1.1	1105
31.5	δ-Cadinene	1,587,040,711	1.5	1112
35.3	γ-Eudesmol	2,559,260,211	5.5	1187

## Data Availability

All data are available on a reasonable request from the corresponding author.
